# The Global Contribution of Outdoor Air Pollution to the Incidence, Prevalence, Mortality and Hospital Admission for Chronic Obstructive Pulmonary Disease: A Systematic Review and Meta-Analysis

**DOI:** 10.3390/ijerph111111822

**Published:** 2014-11-14

**Authors:** Qingkun Song, David C. Christiani, Xiaorong Wang, Jun Ren

**Affiliations:** 1 Beijing Key Laboratory of Cancer Therapeutic Vaccine, Capital Medical University Cancer Center, Beijing Shijitan Hospital, Tie Yi Road 10, Haidian District, Beijing 100038, China; E-Mail: songqingkun@gmail.com; 2 Department of Environmental Health, Harvard School of Public Health, Boston, MA 02115, USA; E-Mail: dchris@hsph.harvard.edu; 3 The Chinese University of Hong Kong, Hong Kong 999077, China; E-Mail: xrwang@cuhk.edu.hk

**Keywords:** ambient air pollution, chronic obstructive pulmonary disease, environmental health, epidemiology, particulate matter

## Abstract

*Objective*: This study aimed to investigate the quantitative effects of outdoor air pollution, represented by 10 µg/m^3^ increment of PM_10_, on chronic obstructive pulmonary disease in China, United States and European Union through systematic review and meta-analysis. *Methods*: Publications in English and Chinese from PubMed and EMBASE were selected. The Cochrane Review Handbook of Generic Inverse Variance was used to synthesize the pooled effects on incidence, prevalence, mortality and hospital admission. *Results*: Outdoor air pollution contributed to higher incidence and prevalence of COPD. Short-term exposure was associated with COPD mortality increased by 6%, 1% and 1% in the European Union, the United States and China, respectively (*p* < 0.05). Chronic PM exposure produced a 10% increase in mortality. In a short-term exposure to 10 µg/m^3^ PM_10_ increment COPD mortality was elevated by 1% in China (*p* < 0.05) and hospital admission enrollment was increased by 1% in China, 2% in United States and 1% in European Union (*p* < 0.05). *Conclusions*: Outdoor air pollution contributes to the increasing burdens of COPD.10 µg/m^3^ increase of PM_10_ produced significant condition of COPD death and exacerbation in China, United States and European Union. Controlling air pollution will have substantial benefit to COPD morbidity and mortality.

## 1. Introduction

Chronic obstructive pulmonary disease (COPD) is one of the leading health burdens worldwide, accounting for 3.0 million deaths annually [1]. Up to 2010, COPD was still the leading cause of deaths, and among the top 5 causes for years of life lost in East and South Asia [2,3]. Globally there are 210 million people suffering from COPD [4]. Without effective intervention, the deaths from COPD will increase by more than 30% in the next decade [4]. The major risk factors of COPD includes tobacco smoking, outdoor air pollution and indoor air pollution from biomass fuel burning [4,5,6]. From some systematic reviews, exposing to indoor air pollution introduced a more than 2-fold risk of COPD, tobacco smoking definitely increased the risk of chronic respiratory diseases and smoking cessation was an effective strategy for COPD treatment [7,8,9,10]. However, the effects from outdoor air pollution on COPD burdens and exacerbation were seldom reported from systematic reviews. Reports of the efficacy of air control strategies on prevention of chronic pulmonary diseases were few. Recently, China faces a huge challenge in outdoor air pollution and chronic respiratory diseases become main issues threatening Chinese people’s health. Verifying the efficacy of air quality improvement on COPD burdens, especially significant reductions of particulate matter (PM), is meaningful to Chinese policymakers. This study aimed to quantitatively assess the contribution from outdoor air pollution to COPD burdens, in order to present a goal and evidence of improving air quality.

## 2. Experimental Section

### 2.1. Data Sources

Databases: PubMed and EMBASE were the target databases. PubMed was accessed through NCBI (from 1January 1980 to 31 March 2012) [11]. EMBASE was accessed via Harvard (from 1980 to 2012) [12]. We selected the publications investigating the effects of outdoor air pollution on COPD, in terms of incidence, prevalence, mortality and hospital admission.

### 2.2. Search Terms and Strategy to Select Articles

Search terms:

(1) Ambient air pollution
(2) Urban air pollution
(3) Outdoor air pollution
(4) COPD
(5) Chronic obstructive pulmonary disease
(6) Chronic bronchitis
(7) Emphysema

In all fields search:

“(1) AND (4)” OR “(1) AND (5)” OR “(1) AND (6)” OR “(1) AND (7)” OR “(2) AND (4)” OR “(2) AND (5)” OR “(2) AND (6)” OR “(2) AND (7)” OR “(3) AND (4)” OR “(3) AND (5)” OR “(3) AND (6)” OR “(3) AND (7)”.

### 2.3. Study Selection

COPD includes chronic bronchitis and emphysema [4]. For the complexity of outdoor air, PM was chosen as the indicator of outdoor air quality [13]. Inclusion criteria were definition of COPD (ICD-10:J41–44), language (Chinese and English), study design (cohort, case-control, cross-sectional and time-series), original reports, relative risk and 95% confidence interval (95%CI) of PM. The exclusion criteria were reanalysis of previous data, review and comments to related study.

### 2.4. Data Extraction and Quality Assessment

The items of publication year, study design, study field, subjects setting, exposure, outcome, effect size, and adjusted factors were all extracted.In case of the collinearity, the extracted effect estimate of PM did not adjust any other pollutants in outdoor air.PM was classified as total suspended particulates (TSP, particulates less than 40 μm in diameter), inhalable particulates (PM_10_, particulates less than 10 μm in diameter), coarse particulates (particulates less than 10 μm but higher than 2.5 μm in diameter), fine particulates (PM_2.5_, particulates less than 2.5 μm in diameter) and ultra-fine particulates (PM_0.1_, particulates less than 0.1 μm in diameter). Most studies investigated PM_10_, and it was set as the representative of PM. Study characteristics of eligible articles were exhibited for quality assessment.

### 2.5. Data Synthesis

Review Manager (RevMan) is The Cochrane Collaboration’s software for preparing and maintaining Cochrane reviews. We used 5.0 version to measure the pooled effect. “Generic Inverse Variance” method was recommended to synthesize data in non-randomized studies [14]. If any studies provided the effect estimate of subgroups, the pooled effect was estimated across subgroups: the procedure can be interpreted as a meta-analysis at the level of an individual study [14]. Most studies investigating the acute effect in 2 days after air quality change, so the average estimate in lag 0–2 day was estimated. In addition, RevMan 5 provided estimates of the heterogeneity. We used Chi^2^ to assess the heterogeneity that *p*-value of Chi^2^ < 0.05 indicated significant heterogeneity. The random analysis model was used for the significant heterogeneity and the fixed analysis model was introduced for non-significant heterogeneity [15]. Publication biases between studies were assessed by funnel plot.

## 3. Results

In total 351 articles were identified by titles and abstracts and 67 were further assessed in details. Finally 44 studies were included in the study (Supplemental Figure S1). The included studies were published after 1990s and most were time-series designs (Supplemental Table S1). Among the studies on mortality, the cohort and cross-sectional study assessed the chronic effect of PM, and the case crossover and time-series studies analyzed the acute effect of PM (less than 7-day exposure) (Supplemental Table S1). Though these studies had adjustments of confounding factors (Supplemental Table S1), the observatory design still introduced median risk of bias. And some publications defined COPD by the symptoms not the lab tests, which was possible to have a high risk of bias.

### 3.1. Outdoor Air Pollution to COPD Incidence

One cohort study in California showed a 1.33-fold high risk of incidence from a more than 200μg/m^3^ TSP exposure. A nested case-control study in Athens, found the risk increased to 1.37 from a quartile increase of black smoke exposure.

### 3.2. Outdoor Air Pollution to COPD Prevalence

**Figure 1:**
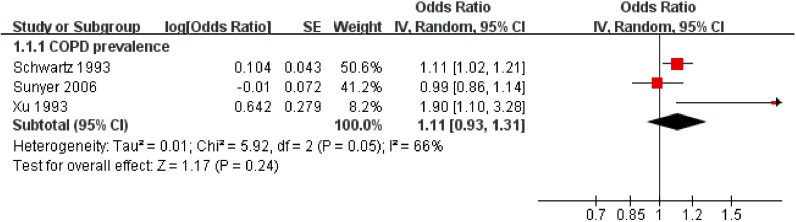
The effect of outdoor air pollution on COPD prevalence.

Three studies investigated the contribution to prevalence. Exposing to high level PM, pooled prevalence risk was increased by 11%, and heterogeneity was not significant (Figure 1).

### 3.3. Outdoor Air Pollution to COPD Mortality

**Figure 2:**
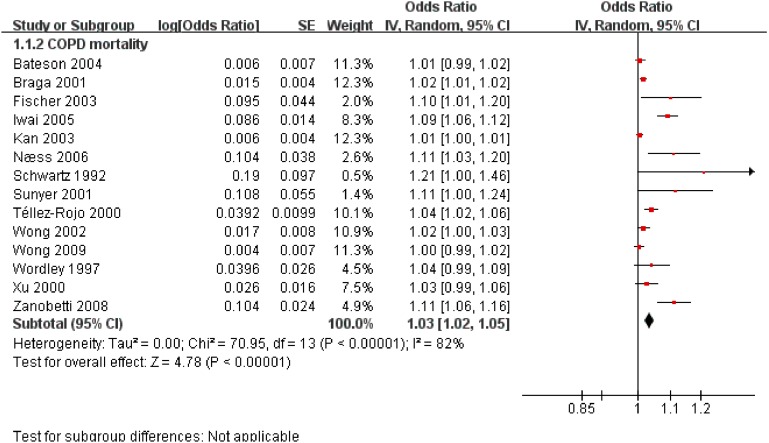
The effect of outdoor air pollution on COPD mortality.

Fourteen studies investigated the contribution to mortality. A 3% higher risk was observed for COPD death but the heterogeneity was significant (Figure 2).

In different study designs, cohort studies observed an 11% higher risk from long-term exposure to exacerbating outdoor air pollution and case crossover studies observed a 1% higher risk in short-term exposure for COPD death (Table 1). In various regions, the pooled risk was 1.07 in EU and 1.01 in China from higher PM exposure; the heterogeneity was non-significant (Table 1). Short-term exposing to 10µg/m^3^ increment of PM_10_ produced a 1% higher acute death in China (Table 1).

**Table 1:** Subgroup analysis for the contribution of outdoor air pollution to COPD mortality *.

Category	Study Characteristics (Number of Studies)	Summary Estimate	Summary Estimate	*I*^2^
Study design	Cohort (2)	1.11	1.07~1.15	0%^#^
	Case crossover (3)	1.01	1.00~1.01	42%^#^
	Ecological study (8)	1.02	1.01~1.03	57%
	Cross sectional (1)	1.09	1.06~1.12	NA
Research field	EU (4)	1.07	1.04~1.11	0%^#^
	US (4)	1.03	1.00~1.06	84%
	China (4)	1.01	1.00~1.01	3%^#^
	others (2)	1.06	1.02~1.11	87%
PM size	PM_10_ (11)	1.02	1.01~1.04	76%
	TSP (3)	1.07	1.01~1.13	79%
PM increment	PM_10_ 10 ug/m^3^ increment (8)	1.02	1.01~1.03	76%
	Others (6)	1.08	1.04~1.12	58%
The effect of 10 ug/m^3^ PM_10_ increment in different areas	China (3)	1.01	1.00~1.01	0%^#^
	US (3)	1.03	1.00~1.06	87%
	EU (1)	1.04	0.99~1.06	NA
	Others (1)	1.04	1.02~1.06	NA
Effect duration	Chronic effect (3)	1.10	1.07~1.12	0%^#^
	Acute effect (11)	1.02	1.01~1.03	59%
Acute effects in different areas	China (4)	1.01	1.00~1.01	3%^#^
	EU (3)	1.06	1.02~1.11	0%^#^
	US (3)	1.01	1.01~1.02	56%^#^

* The significance level for heterogeneity was 0.05; ^#^ non-significant.

Based on exposure term, long-term exposure (chronic effect) resulted in a 10% increase for mortality and short-term exposure (acute effect) introduced the death increased by 1% higher in China, 6% higher in EU and 1% higher in US (Table 1). 75% studies in China and US analyzed the acute effect of 10 µg/m^3^ PM_10_ increase, in contrast with 25% studies in EU. The other studies in EU investigated the chronic effect and the acute effect of more than 20 µg/m^3^ PM_10_ increase.

### 3.4. Outdoor Air Pollution and Hospital Admission for COPD

**Figure 3:**
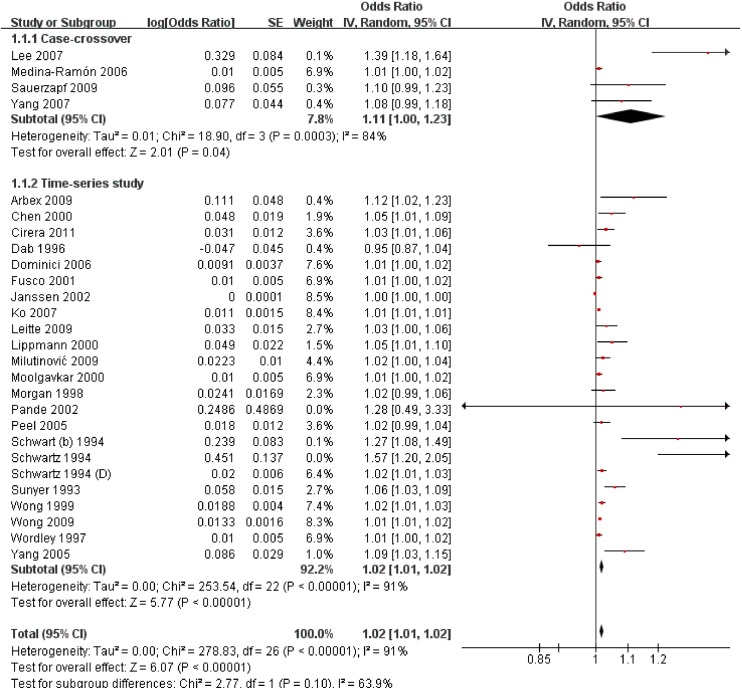
The effect of outdoor air pollution on COPD hospital admission.

From Figure 3, the included studies presented the acute effect of exposure and the total effect estimate of hospital admissions was 1.02 with 95% CI: 1.01–1.02. The subgroup analysis by study design, research fields and PM size did not reduce the heterogeneities; but short-term exposing to10 ug/m^3^ increment of PM_10_ led to the hospital admission increased by 1% in China, 2% in US and 1% in EU, and the heterogeneity was non-significant (Table 2).

### 3.5. Publication Bias

The publication bias was tested by funnel plots among the time-series studies about mortality and all the studies about hospital admission (Supplemental Figures S2 and S3). For mortality, the missing studies appeared in areas of 0.05 < *p* < 0.10; for hospital admission, the supposed missing studies were in area of *p* < 0.05. And when we funnel plotted the studies about hospital admission of 10 μg/m^3^ increase of PM_10_, almost all study plots were on the effect estimate line. Most missing studies were in the significant level, so the asymmetry of funnel plots was probably not caused by publication bias.

**Table 2:** Subgroup analysis for the contribution of outdoor air pollution to hospital admission for COPD *.

Category	Study Characteristics (Number of Studies)	Summary Estimate	Summary Estimate	*I*^2^
Study Design	Case-crossover (4)	1.11	1.00 ~ 1.23	84%
	Ecological study (23)	1.02	1.01 ~ 1.02	64%
Research Field	US (10)	1.02	1.01 ~ 1.03	71%
	EU (8)	1.02	1.01 ~ 1.04	61%
	China (5)	1.01	1.01 ~ 1.02	80%
	Others (4)	1.05	1.02 ~ 1.08	46% ^#^
PM size	PM_10_ (19)	1.02	1.01 ~ 1.02	72%
	Others (8)	1.02	1.01 ~ 1.03	60%
PM increment	PM_10_ 10 ug/m^3^ increment (9)	1.01	1.01 ~ 1.01	16% ^#^
	Others (18)	1.03	1.02 ~ 1.05	75%
The effect of 10 ug/m^3^ PM_10_ increment in different areas	china (3)	1.01	1.01 ~ 1.01	46% ^#^
	US (4)	1.02	1.01 ~ 1.03	0% ^#^
	EU (2)	1.01	1.00 ~ 1.02	59% ^#^

* The significant level for heterogeneity was 0.05; ^#^ non-significant.

## 4. Discussion

Smoking and indoor air pollution introduced increasing risk to COPD. In this study, PM exposures from outdoor air pollution definitively increased COPD burdens. Ten μg/m^3^ increase of PM_10_ was related with higher risk of COPD death and hospital admission.

PM exposure could induce biological changes in respiratory system. PM suspensions increased airway hyper-responsiveness to acetylcholine and reduced host defense in rodents; the exposure released neutrophil influx, bronchoalveolar lavage protein and cytokine in lung tissues [16]. Ambient air particles could induce the production of reactive oxygen and inflammatory factors in alveolar macrophages [17,18,19], polymorphonuclear granulocytes [20] and bronchial epithelial cells [21]. The reactive oxygen species, inflammatory factor production and respiratory inflammation, played important roles in lung tissue injury and higher risk of COPD.

The relative risk for incidence was observed higher than prevalence. The studies on COPD incidence investigated TSP of higher than 200μg/m^3^ [22] and black smoke of more than 10 μg/m^3^ increase [23] but the studies on COPD prevalence analyzed PM_2.5_ of 1 μg/m^3^ increase [24] and TSP of 10 μg/m^3^ increase [25]. Higher exposure might introduce a higher effect. Additionally, PM increase in outdoor air pollution was associated with a higher risk of death, and the severe cases might die due to the exposure and old age. Therefore, a lower effect estimate was observed in prevalent cases than incident cases. The stronger contribution to prevalence in China than Western countries is likely related to the severe air quality in China [26].

The acute effect of mortality was a little higher in EU than US and China, which was the result of different exposure characteristics: the studies in China and US analyzed the effect of lower level exposure than the studies in EU. The chronic effect of outdoor air pollution on COPD mortality was much more severe than acute effect, because of longer exposure time.The increase of hospital admission for COPD after the outdoor air pollution rise suggested that outdoor air pollution affected the exacerbation of COPD status.

When a funnel plot was used to detect the publication bias in this analysis, it seemed that the plots were somewhat visually asymmetrical. The asymmetry indicated there were small-study effects in the analysis [14]. Though some statistical methods were recommended by the Cochrane center for testing the asymmetry of funnel plot, the methods had relatively low power and were recommended in randomized control trials [14]. Therefore, we chose contour enhanced funnel plot to test the publication bias [14]. For the missing studies in significant areas, the asymmetry of funnel plot might not be the result of publication bias, but the heterogeneity and the artefactual: the association between effect estimate and standard error [14].

The inclusion of non-randomized studies was the primary limitation in this study. The Handbook Review of Cochrane still provided analytical methods for non-randomized studies. As suggested from Cochrane, generic inverse variation was selected to synthesize the data from non-randomized studies and the study characteristics were presented [14]. In contrast to randomized control trials, confounding bias was one of the key issues in non-randomized studies.

The primary risk factors for COPD were tobacco smoking, indoor air pollution (such as biomass fuels), outdoor air pollution and occupational dusts and chemicals [4], and most of the included studies controlled these risk factors. Two cohorts failed to control the effects of tobacco smoking, but smoking appeared not to act as a confounder in analyses [27,28]. The case crossover and time series studies analyzed the acute effects of outdoor air pollution on COPD and in such a short term, smoking habit and biomass fuel usage were not possible to change seriously. Misclassification of exposure and outcome was another possible limitation in this analysis. In the included studies, the data of exposure to PM was provided by the government environmental department, who monitored the outdoor air quality and the diagnosis of COPD was defined in hospitals. The non-random design and potential risk of bias introduced the limitation in this study.

## 5. Conclusions

High level outdoor air pollution was associated with an increase of COPD incidence and prevalence. A 10 μg/m^3^ increase of PM_10_ in outdoor air can induce significant acute exacerbations and mortality in COPD. This study provides evidence for the need for air quality improvement and continual assessment. It is time to take action to improve air quality.

## Supplementary Files

Supplementary File 1

## Strengths

This study integrated the studies in both English and Chinese and presented the effect of PM pollution in Western Countries and China. Besides of the significant effect of PM on COPD burden, it showed the contribution of 10µg/m^3^ increased to COPD death and exacerbation. It estimated the acute and chronic effect of PM increase on COPD death.

It provided a quantitative evidence for government, especially for China to set up an air quality plan to achieve an acceptable goal in COPD control.

## Limitations

It included non-randomized studies. Most of the included studies were ecological design, which did not control the risk factors of smoking and indoor air pollution.
