# Supplementary material for: The Global Contribution of Outdoor Air Pollution to the Incidence, Prevalence, Mortality and Hospital Admission for Chronic Obstructive Pulmonary Disease: A Systematic Review and Meta-Analysis
Source: Int J Environ Res Public Health. 2014 Nov 14;11(11):11822–32. doi: 10.3390/ijerph111111822 (PMC4245645; doi:10.3390/ijerph111111822)
Supplement: Supplementary File 1 [file ijerph-11-11822-s001.pdf]

Supplementary Information

The Global Contribution of Outdoor Air Pollution to the Incidence, Prevalence, Mortality and Hospital Admission for Chronic Obstructive Pulmonary Disease: A Systematic Review and Meta-Analysis

Table S1. The study characteristics for included articles.

Publication Year	Study Design	Research Field	Settings	Exposure	Outcome	Effect size	adjusted factors
1991 [1]	Cohort	California	no smoking California Seventh-Day Adventists were monitored for a 6-year period,	TSP	New cases of definite symptoms of chronic bronchitis	Significant association was observed	Age, education, sex, childhood colds, childhood air obstructive disease, possible symptoms, years smoked, years lived with a smoker, and years worked with a smoker.
2003 [2]	Nested case-control study	Athens	84 cases and 168 healthy controls	Black Smoke	Incidence of COPD	Significant association was observed	Age, gender, smoking habits and education
2006 [3]	Cohort	21 cities in 10 EU countries	Randomized subjects from 21 cities	$PM_{2.5}$	prevalence chronic bronchitis	No significant association was observed	Age, Smoking status, respiratory infections, rhinitis, social class and traffic
1993 [4]	cross-sectional study	53 USurban areas	Representative sample of US population	TSP	Prevalence of Chronic Bronchitis	Significant association was observed	Age, race, sex and smoking
1993 [5]	cross-sectional study	Beijing	1576 subjects with smoking status in age of 40–69	TSP	Prevalence of chronic bronchitis	Significant association was observed	Income, indoor crowding, occupational exposure, cooking gases or fumes, indoor coal combustion and passive smoking

Table S1. *Cont.*

Publication Year	Study Design	Research Field	settings	Exposure	Outcome	Effect size	adjusted factors
2008 [6]	Cohort	34 cities in US	COPD cases	PM_{10}	COPD death	Significant association was observed for PM_{10}	Age, gender, race, Season of admission, number of days of coronary and medical intensive care, previous diagnoses for some diseases, time period and season
2007 [7]	Cohort	Oslo, Norway	All inhabitants of Oslo, Norway, aged 51–90 years on 1992	PM_{10} and $PM_{2.5}$	COPD death	Both increased COPD mortality in both genders	occupational class and length of education
2004 [8]	case-crossover study	Cook County, Illinois	Elderly residents with a history of hospitalization for heart or lung disease	PM_{10}	COPD death	No significant association was observed	Mean temperature, humidity, and barometric pressure
2001 [9]	case-crossover study	Barcelona	Residents > 35 years, attended emergency room for COPD 1985–1989 and died 1990–1995	PM_{10}	COPD death	PM_{10} increase associated with the higher mortality of COPD	Temperature, humidity, hot days and influenza days
2003 [10]	case-crossover study	Shanghai	Data of cause-specific mortality and air pollution 2000–2001	PM_{10}	COPD death	No significant association was observed	Mean temperature and humidity

Table S1. *Cont.*

Publication Year	Study Design	Research Field	Settings	Exposure	Outcome	Effect Size	Adjusted Factors
2001 [11]	Time-series study	10 US cities	Subjects from 10 cities of US	PM_{10}	COPD death	Significant association was observed	Weather and season
1992 [12]	Time-series study	Philadelphia	Daily death from 1973–1980	TSP	COPD death	Significant association was observed	Year, season, temperature, humidity
2000 [13]	Time-series study	Mexico	Mortality data of Mexico city population	PM_{10}	COPD death	Increased mortality associated with PM	time, month, temperature, relative humidity, day of the week and holidays
2003 [14]	Time-series study	Netherland	Mortality counts from 1986–1994	PM_{10} and Black Smoke	COPD death	In some age-specific groups, the significant association was observed	Long-term trends, seasonal trends, influenza epidemics, ambient temperature, ambient relative humidity, day of the week and holidays
1997 [15]	Time-series study	Birmingham	Mortality data of Birmingham population 1992–1994	PM_{10}	COPD death	The significant association existed in some lag day	Temperature and relative humidity
2005 [16]	cross-sectional study	Japan	The annual statistics and air pollution estimates throughout Japan	PM_{10} and $PM_{2.5}$	COPD death	In females, a significant association was observed	Age and smoking rate

Table S1. *Cont.*

Publication Year	Study Design	Research Field	Settings	Exposure	Outcome	Effect Size	Adjusted Factors
2002 [17]	Time-series study	Hong Kong	Daily mortalities for respiratory and cardiovascular diseases 1995–1998	PM_{10}	COPD death	Significant association was observed	Day of the time series, days of the week, seasonal variations, temperature and humidity
2009 [18]	Time-series study	Hong Kong	Daily counts of hospitalization and mortalities from the 14 general hospitals 1996–2002	PM_{10}	COPD death	No significant association was observed	Daily mean temperature, relative humidity and influenza
2000 [19]	Time-series study	Shenyang, China	Air pollution in 1992 and daily mortality data of 1992	TSP	COPD death	No significant association was observed	Temperature, humidity, and Sunday
2002 [20]	Time-series study	14 cities in US	Persons ≥ 65 years from 14 cities and daily PM_{10} measurements 1985–1994	PM_{10}	Hospital admission of COPD	No significant association was observed	Season, weather variables (24-h means of temperature, relative humidity, and barometric pressure) and day of week
2006 [21]	Time-series study	204 US urban counties	National database comprising daily time-series data 1999–2002	$PM_{2.5}$	Hospital admissions for COPD	Significant association was observed	Temperature and dew-point temperature

Table S1. *Cont.*

Publication Year	Study Design	Research Field	Settings	Exposure	Outcome	Effect Size	Adjusted Factors
2000 [22]	Time-series study	3 counties in US	Daily hospital admissions 1987–1995	PM_{10} and $PM_{2.5}$	Hospital admission of COPD	Significant association was observed	Temporal trends, temperature, relative humidity, and day of week
2000 [23]	Time-series study	Nevada	Daily hospital admissions 1990–1994.	PM_{10}	Hospital admission of COPD	Significant association was observed	Weather variables, day of week, seasons, and time trend
1994 [24]	Time-series study	Birmingham, Alabama	Records for pneumonia and COPD, 1986–1989	PM_{10}	Hospital admissions for COPD	Significant association existed	Time trends, seasonal fluctuations, and weather
1994 [25]	Time-series study	Minneapolis, Minnesota	Medicare records for the years 1986 through 1989.	PM_{10}	Hospital admissions for COPD	In lag 0–1 day, the significant association was observed	Temperature, dew point temperature and time terms
2005 [26]	Time-series study	Atlanta	Hospital admission of COPD 1993–2000	PM_{10}, coarse PM & $PM_{2.5}$	Hospital admissions for COPD	Non-significant association existed in lag 0–2 day	Day-of-week, hospital entry/exit, and holidays, time with monthly knots, season indicator variables,temperature and dew point temperature
1994 [27]	Time-series study	Detroit, Michigan	Hospital admission of COPD 1986–1989	PM_{10}	Hospital admission of COPD	Significant association existed	Seasonal, temporal trends, temperature and dew point temperature
2000 [28]	Time-series study	Michigan	Hospital admission of COPD 1982–1994	PM_{10}	Hospital admission of COPD	Significant association was observed in the lag 3 day	Temperature, humidity, seasonal cycles and influenza epidemics

Table S1. *Cont.*

Publication Year	Study Design	Research Field	Settings	Exposure	Outcome	Effect Size	Adjusted Factors
2005 [29]	Time-series study	Vancouver, British Columbia, Canada	Dailycounts of acute COPD hospitalization 1994–1998	PM_{10}	Hospital admissions for COPD	Significant association was observed	Weather conditions
1997 [15]	Time-series study.	Birmingham, United Kingdom	Air pollution data were taken from a national network monitoring station 1992–1994	PM_{10}	Hospital admissions for COPD	Non-significant association existed	Day of the week and month, Maximum daily temperature and mean daily relative humidity
1993 [30]	Time-series study	Barcelona	Daily emergency room admissions for residents ≥ 14 years with COPD, 1985–1989	Black smoke	Hospital admissions for COPD	Significant association existed in winter	Temperature, day of the week, and year
1996 [31]	Time-series study	Paris	Hospital admission for COPD, 1987–1992	Black smoke and PM_{13}	Hospital admission for COPD	Non-significant association was observed	Linear trend, , day of the week, influenza A epidemic, holidays, temperature, humidity
2001 [32]	Time-series study	Rome	Emergency hospital admissions for respiratory conditions, 1995–1997	TSP	hospital admissions for COPD	Non-significant association was observed	The day of study, mean temperature, mean humidity, influenza epidemics, and indicator variables for day of the week and holidays

Table S1. *Cont.*

Publication Year	Study Design	Research Field	Settings	Exposure	Outcome	Effect Size	Adjusted Factors
2009 [33]	Time-series study	Drobeta-Turnu Severin, Romania	Hospital admissions of chronic bronchitis and COPD in 586 days	TSP	Hospital admissions for COPD and chronic bronchitis	Significant association was observed	Day of the week and holiday and nonlinear effects of time, of temperature, of infectious diseases and humidity
2009 [34]	Time-series study	Nis, Serbia	Daily counts of emergency room visits and air pollution in 2002	Black smoke	Emergency room visit for COPD	In lag 0–2 day, significant association was observed	Time trend, seasonal variation, days of week, temperature, relative humidity air pressure, precipitation, rainfall, snowfall and wind
1999 [35]	Time-series study	Hong Kong	Hospital admission of COPD in Hong Kong 1994–1995	PM_{10}	Hospital admissions for COPD	In lag 0–3 day, significant association was observed	Trend, season, and other cyclical factors, temperature, and humidity
2007 [36]	Time-series study.	Hong Kong	Hospital admission for COPD and air condition in HK 2000–2004	PM_{10} and $PM_{2.5}$	Hospital admission for COPD	Significant association could be got in some lag day	Time trend, season, other cyclical factors, temperature and humidity
2009 [18]	Time-series study	Hong Kong	Hospitalization from the 14 general hospitals, 1996–2002	PM_{10}	Hospital admission for COPD	Significant association was observed in lag 0–1 day	Daily mean temperature, relative humidity and influenza

Table S1. *Cont.*

Publication Year	Study Design	Research Field	Settings	Exposure	Outcome	Effect Size	Adjusted Factors
1998 [37]	Time-series study	Sydney	Hospital admission of COPD 1990–1994	PM_{10}	Hospital admissions for COPD	There was a non-significant association in lag 0 day	Weather, seasonal trends and temperature
2002 [38]	Time-series study	Delhi	Daily emergency room visits and air pollution data 1997–1998	TSP	Emergency room visits for COPD	Non-significant association existed	Season, day of the week, temperature and humidity
2011 [39]	Time-series study	Cartagena	Daily emergency room visits and air pollution data 1995–1998	TSP	Emergency room visits for COPD	Significant associations were observed in lag 0 day	Season, weather indicators, influenza, day of the week, andpublic holidays
2009 [40]	Time-series study	Sa˜o Paulo	COPD emergency room visits and air pollution data 2001–2003	PM_{10}	Emergency room visits for COPD	Significant associations were observed in lag 0–2 days	Season, temperature and humidity
2009 [41]	case crossover study	England	Daily COPD admissions were recorded, 2006–2007	PM_{10}	Daily COPD admissions	Non-significant association existed in the mean 8 days	Maximum temperature, pollen, and influenza infection.

Table S1. *Cont.*

Publication Year	Study Design	Research Field	Settings	Exposure	Outcome	Effect Size	Adjusted Factors
2007 [42]	case crossover study	Taipei, Taiwan	Hospital admissions for COPD and ambient air pollution data for Taipei 1996–2003	PM_{10}	Hospital admissions for COPD	In higher temperature, a significant association was observed	Temperature and humidity.
2007 [43]	case crossover study	Kaohsiung, Taiwan	Hospital admissions for COPD and ambient air pollution data for Kaohsiung 1996–2003	PM_{10}	Hospital admissions for COPD	Significant association existed	Temperature and humidity.
2006 [44]	case crossover study	36 US cities	Respiratory hospital admissions and air pollution data 1986–1999	PM_{10}	Hospital admission for COPD	In warm season, there was a significant association	Day of the week and weather

Figure S1. Flow diagram for study search, inclusions and exclusions.

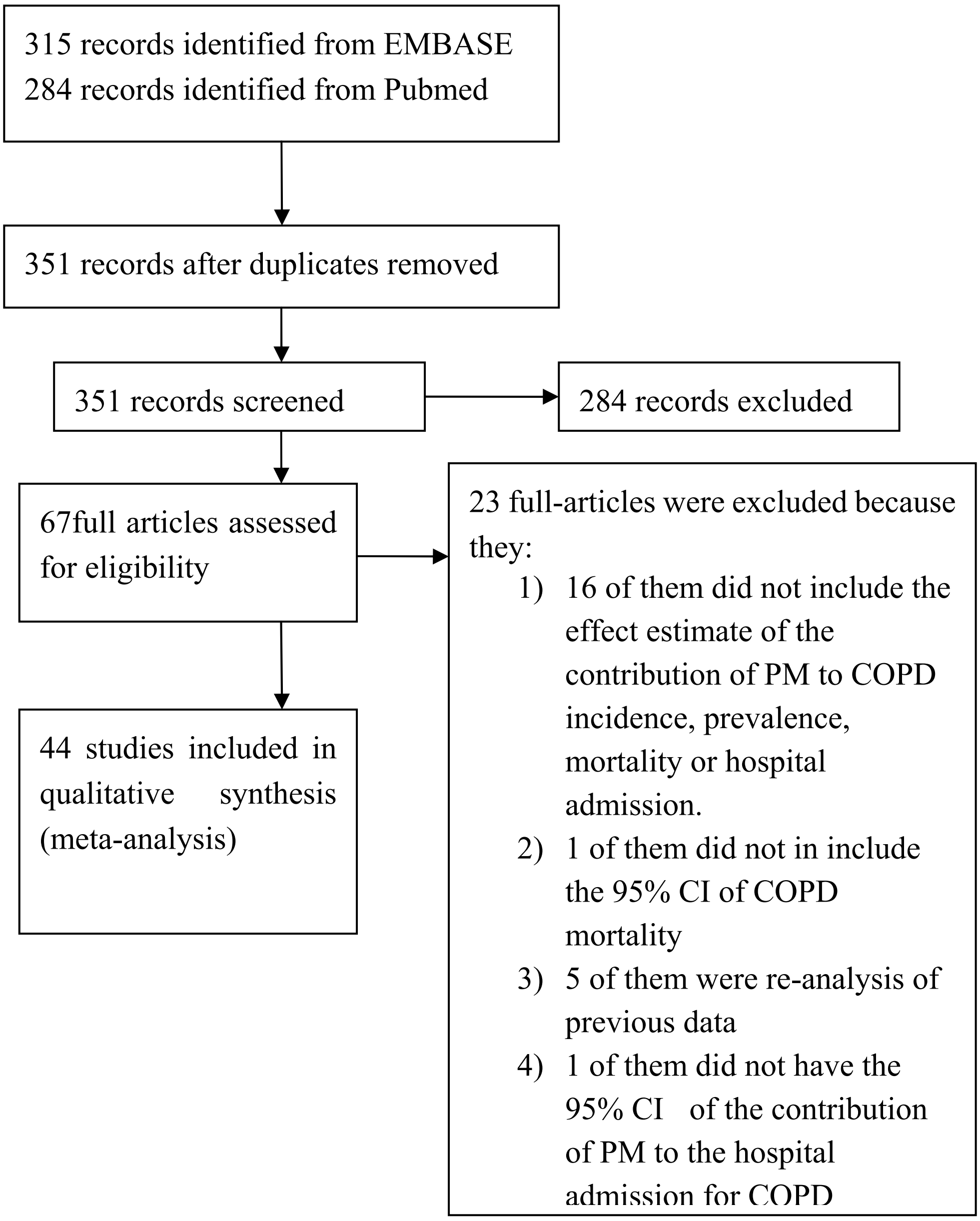

Figure S2. Funnel plot for the studies on COPD mortality.

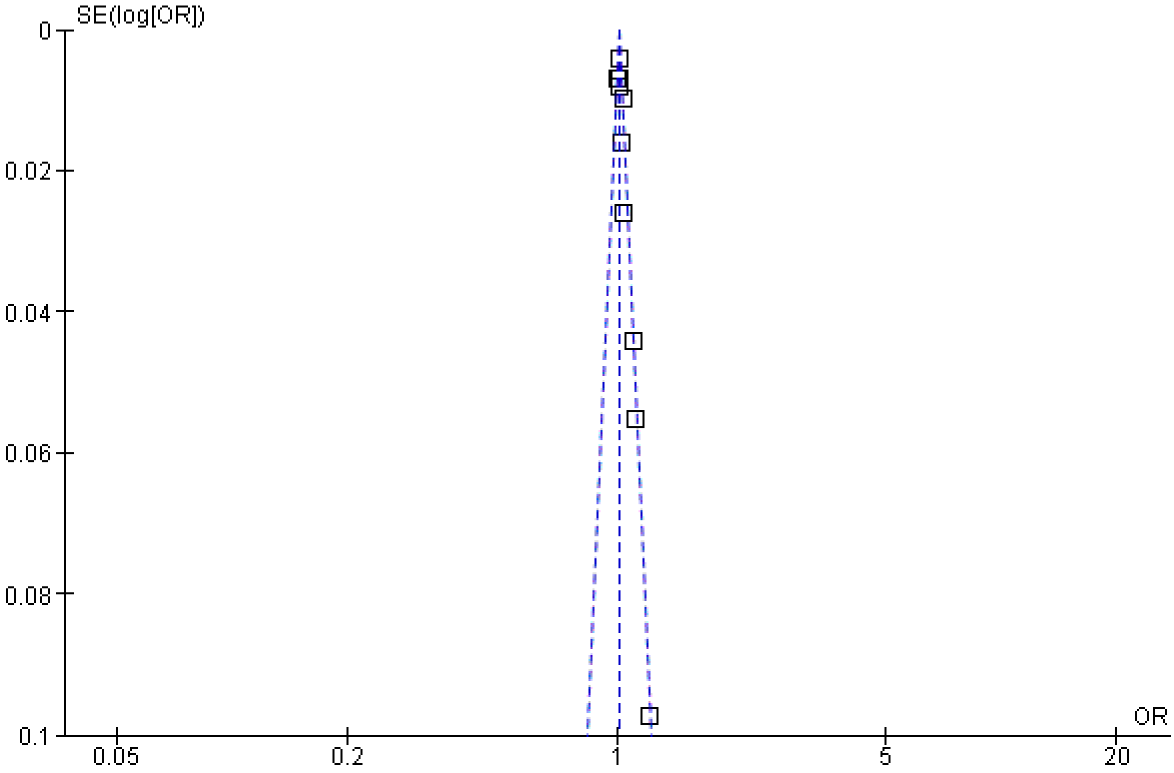

Figure S3. Funnel plot for the studies about hospital admission for COPD.

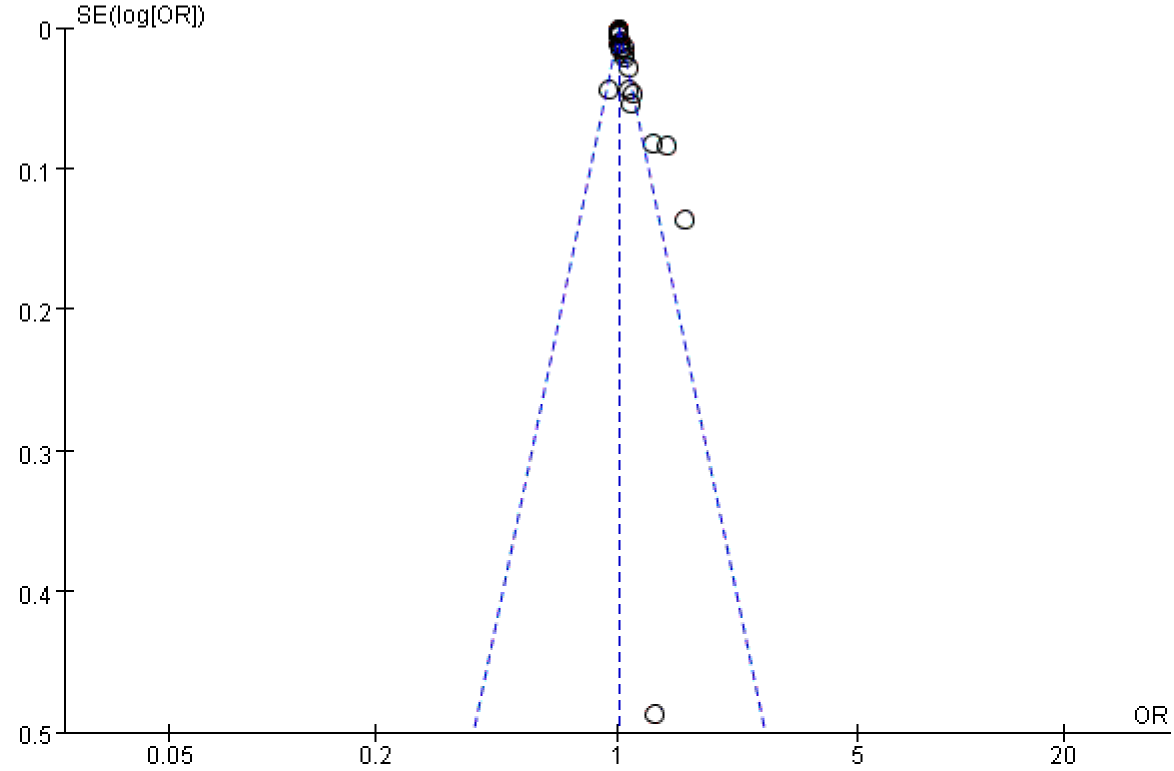

References

1. Abbey, D.E.; Mills, P.K.; Petersen, F.F.; Beeson, W.L. Long-Term ambient concentrations of total suspended particulates and oxidants as related to incidence of chronic disease in california seventh-day adventists. *Environ. Health Perspect.* **1991**, *94*, 43–50.
2. Karakatsani, A.; Andreadaki, S.; Katsouyanni, K.; Dimitroulis, I.; Trichopoulos, D.; Benetou, V.; Trichopoulou, A. Air pollution in relation to manifestations of chronic pulmonary disease: A nested case-control study in Athens, Greece. *Eur. J. Epidemiol.* **2003**, *18*, 45–53.
3. Sunyer, J.; Jarvis, D.; Gotschi, T.; Garcia-Esteban, R.; Jacquemin, B.; Aguilera, I.; Ackerman, U.; Marco, R.D.; Forsberg, B.; Gislason, T.; Heinrich, J.; Norbäck, D.; Villani, S.; Künzli, N. Chronic bronchitis and urban air pollution in an international study. *Occup. Environ. Med.* **2006**, *63*, 836–843.
4. Schwartz, J. Particulate air pollution and chronic respiratory disease. *Environ. Res.* **1993**, *62*, 7–13.
5. Xu, X.; Wang, L. Association of indoor and outdoor particulate level with chronic respiratory illness. *Am. Rev. Respir. Dis.* **1993**, *148*, 1516–1522.
6. Zanobetti, A.; Bind, M.-A.C.; Schwartz, J. Particulate air pollution and survival in a COPD cohort. *Environ. Health* **2008**, *7*, doi:10.1186/1476-069X-7-48.
7. Næss, Ø.; Nafstad, P.; Aamodt, G.; Claussen, B.; Rosland, P. Relation between concentration of air pollution and cause-specific mortality: Four-year exposures to nitrogen dioxide and particulate matter pollutants in 470 neighborhoods in Oslo, Norway. *Am. J. Epidemiol.* **2007**, *165*, 435–443.
8. Bateson, T.F.; Schwartz, J. Who is sensitive to the effects of particulate air pollution on mortality? A case-crossover analysis of effect modifiers. *Epidemiology* **2004**, *15*, 143–149.
9. Sunyer, J.; Basagaña, X. Particles, and not gases, are associated with the risk of death in patients with chronic obstructive pulmonary disease. *Int. J. Epidemiol.* **2001**, *30*, 1138–1140.
10. Kan, H.; Chen, B.; Jia, J. A case-crossover study of ambient air pollution and daily mortality in Shanghai. *Zhonghua Liu Xing Bing Xue Za Zhi* **2003**, *24*, 863–867.
11. Braga, A.L.F.; Schwartz, J. The lag structure between particulate air pollution and respiratory and cardiovascular deaths in 10 US cities. *J. Occup. Environ. Med.* **2001**, *43*, 927–933.
12. Schwartz, J.; Dcokery, D.W. Increased mortality in Philadelphia associated with daily air pollution concentrations. *Am. Rev. Respir. Dis.* **1992**, *145*, 600–604.
13. Téllez-Rojo, M.M.; Romieu, I.; Ruiz-Velasco, S.; Lezana, M.A.; Hernández-Avila, M.M. Daily respiratory mortality and PM_{10} pollution in Mexico City: Importance of considering place of death. *Eur. Respir. J.* **2000**, *16*, 391–396.
14. Fischer, P.; Hoek, G.; Brunekreef, B.; Verhoeff, A.; Wijnenz, J.V., Air pollution and mortality in The Netherlands: Are the elderly more at risk? *Eur. Respir. J.* **2003**, *21*, 34s–38s.
15. Wordley, J.; Walters, S.; Ayres, J.G. Short term variations in hospital admissions and mortality and particulate air pollution. *Occup. Environ. Med.* **1997**, *54*, 108–116.
16. Iwai, K.; Mizuno, S.; Miyasaka, Y.; Mori, T. Correlation between suspended particles in the environmental air and causes of disease among inhabitants: Cross-sectional studies using the vital statistics and air pollution data in Japan. *Environ. Res.* **2005**, *99*, 106–117.

17. Wong, T.W.; Tam, W.S.; Yu, T.S.; Wong, A.H.S. Associations between daily mortalities from respiratory and cardiovascular diseases and air pollution in Hong Kong, China. *Occup. Environ. Med.* **2002**, *59*, 30–35.
18. Wong, C.M.; Yang, L.; Thach, T.Q.; Chau, P.Y.K.; Chan, K.P.; Thomas, G.N.; Lam, T.H.; Wong, T.W.; Hedley, A.J.; Peiris, J.S.M. Modification by influenza on health effects of air pollution in Hong Kong. *Environ. Health Perspect.* **2009**, *117*, 248–253.
19. Xu, Z.; Yu, D.; Jing, L.; Xu, X. Air pollution and daily mortality in Shenyang, China. *Arch. Environ. Health* **2000**, *55*, 115–120.
20. Janssen, N.A.H.; Schwartz, J.; Zanobetti, A.; Suh, H.H. Air conditioning and source-specific particles as modifiers of the effect of PM_{10} on hospital admissions for heart and lung disease. *Environ. Health Perspect.* **2002**, *110*, 43–49.
21. Dominici, F.; Peng, R.D.; Bell, M.L.; Pham, L.; McDermott, A.; Zeger, S.L.; Samet, J.M. Fine particulate air pollution and hospital admission for cardiovascular and respiratory diseases. *JAMA* **2006**, *295*, 1127–1134.
22. Moolgavkar, S.H. Air pollution and hospital admissions for chronic obstructive pulmonary disease in three metropolitan areas in the United States. *Inhal. Toxicol.* **2000**, *12*, 75–90.
23. Chen, L.; Yang, W.; Jennison, B.L.; Omaye, S.T. Air particulate pollution and hospital admissions for chronic obstructive pulmonary disease in Reno, Nevada. *Inhal. Toxicol.* **2000**, *12*, 281–298.
24. Schwartz, J. Air pollution and hospital admissions for the elderly in Birmingham, Alabama. *Am. J. Epidemiol.* **1994**, *139*, 589–598.
25. Schwartz, J. PM_{10}, ozone, and hospital admissions for the elderly in Minneapolis-St. Paul, Minnesota. *Arch. Environ. Health* **1994**, *49*, 366–374.
26. Peel, J.L.; Tolbert, P.E.; Klein, M.; Metzger, K.B.; Flanders, W.D.; Todd, K.; Mulholland, J.A.; Ryan, P.B.; Frumkin, H. Ambient air pollution and respiratory emergency department visits. *Epidemiology* **2005**, *16*, 164–174.
27. Schwartz, J. Air pollution and hospital admissions for the elderly in Detroit, Michigan. *Am. J. Respir. Crit. Care Med.* **1994**, *150*, 648–655.
28. Lippmann, M.; Ito, K.; Nádas, A.; Burnett, R.T. Association of particulate matter components with daily mortality and morbidity in urban populations. *Res. Rep. Health Effect Inst.* **2000**, *95*, 5–72.
29. Yang, Q.Y.; Chen, Y.; Krewski, D.; Burnett, R.T.; Shi, Y.L.; McGrail, K.M. Effect of short-term exposure to low levels of gaseous pollutants on chronic obstructive pulmonary disease hospitalizations. *Environ. Res.* **2005**, *99*, 99–105.
30. Sunyer, J.; Sáez, M.; Murillo, C.; Castellsague, J.; Martínez, F.; Antó, J.M. Air pollution and emergency room admissions for chronic obstructive pulmonary disease: A 5-year study. *Am. J. Epidemiol.* **1993**, *137*, 701–705.
31. Dab, W.; Medina, S.; Quénel, P.; Moullec, Y.L.; Tertre, A.L.; Thelot, B.; Monteil, C.; Lameloise, P.; Pirard, P.; Momas, I.; Ferry, R.; Festy, B. Short term respiratory health effects of ambient air pollution: Results of the APHEA project in Paris. *J. Epidemiol. Community Health* **1996**, *50*, 42s–46s.
32. Fusco, D.; Forastiere, F.; Michelozzi, P.; Spadea, T.; Ostro, B.; Arcà, M.; Perucci, C.A. Air pollution and hospital admissions for respiratory conditions in Rome, Italy. *Eur. Respir. J.* **2001**, *17*, 1143–1150.

33. Leitte, A.M.; Petrescu, C.; Franck, U.; Richter, M.; Suciu, O.; Ionovici, R.; Herbarth, O.; Schlink, U. Respiratory health, effects of ambient air pollution and its modification by air humidity in Drobeta-Turnu Severin, Romania. *Sci. Total Environ.* **2009**, *407*, 4004–4011.
34. Milutinović, S.; Nikić, D.; Stosić, L.; Stanković, A.; Bogdanović, D. Short-term association between air pollution and emergency room admissions for chronic obstructive pulmonary disease in Nis, Serbia. *Cent. Eur. J. Public Health* **2009**, *17*, 8–13.
35. Wong, T.W.; Lau, T.S.; Yu, T.S.; Neller, A.; Wong, S.L.; Tam, W.; Pang, S.W. Air pollution and hospital admissions for respiratory and cardiovascular diseases in Hong Kong. *Occup. Environ. Med.* **1999**, *56*, 679–683.
36. Ko, F.W.S.; Tam, W.; Wong, T.W.; Chan, D.P.S.; Tung, A.H.; Lai, C.K.W.; Hui, D.S.C. Temporal relationship between air pollutants and hospital admissions for chronic obstructive pulmonary disease in Hong Kong. *Thorax* **2007**, *62*, 780–785.
37. Morgan, G.; Corbett, S.; Wlodarczyk, J. Air pollution and hospital admissions in Sydney, Australia, 1990 to 1994. *Am. J. Public Health* **1998**, *88*, 1761–1766.
38. Pande, J.N.; Bhatta, N.; Biswas, D.; Pandey, R.M.; Ahluwalia, G.; Siddaramaiah, N.H.; Khilnani, G.C. Outdoor air pollution and emergency room visits at a hospital in Delhi. *Indian J. Chest Dis. Allied Sci.* **2002**, *44*, 13–19.
39. Cirera, L.; Garcia-Marcos, L.; Gimenez, J.; Moreno-Grau, S.; Tobias, A.; Perez-Fernandez, V.; Elvira-Rendeles, B.; Guillen, J.J.; Navarro, C. Daily effects of air pollutants and pollen types on asthma and COPD hospital emergency visits in the industrial and Mediterranean Spanish city of Cartagena. *Aller. Immunopathol. (Madr.)* **2011**, *40*, 231–237.
40. Arbex, M.A.; de Souza Conceicao, G.M.; Cendon, S.P.; Arbex, F.F.; Lopes, A.C.; Moyses, E.P.; Santiago, S.L.; Saldiva, P.H.; Pereira, L.A.; Braga, A.L. Urban air pollution and chronic obstructive pulmonary disease-related emergency department visits. *J. Epidemiol. Community Health* **2009**, *63*, 777–783.
41. Sauerzapf, V.; Jones, A.P.; Cross, J. Environmental factors and hospitalisation for chronic obstructive pulmonary disease in a rural county of England. *J. Epidemiol. Community Health* **2009**, *63*, 324–328.
42. Yang, C.Y.; Chen, C.J. Air pollution and hospital admissions for chronic obstructive pulmonary disease in a subtropical city: Taipei, Taiwan. *J. Toxicol. Environ. Health* **2007**, *70*, 1214–1219.
43. Lee, I.M.; Tsai, S.S.; Chang, C.C.; Ho, C.K.; Yang, C.-Y. Air pollution and hospital admissions for chronic obstructive pulmonary disease in a tropical city: Kaohsiung, Taiwan. *Inhal. Toxicol.* **2007**, *19*, 393–398.
44. Medina-Ramón, M.; Zanobetti, A.; Schwartz, J. The effect of ozone and PM_{10} on hospital admissions for pneumonia and chronic obstructive pulmonary disease: A national multicity study. *Am. J. Epidemiol.* **2006**, *163*, 579–588.

© 2014 by the authors; licensee MDPI, Basel, Switzerland. This article is an open access article distributed under the terms and conditions of the Creative Commons Attribution license (http://creativecommons.org/licenses/by/4.0/).
